# Optical Interferometric Device for Rapid and Specific Detection of Biological Cells

**DOI:** 10.3390/bios14090421

**Published:** 2024-08-29

**Authors:** Sándor Valkai, Dániel Petrovszki, Zsombor Fáskerti, Margaréta Baumgärtner, Brigitta Biczók, Kira Dakos, Kevin Dósa, Berill B. Kirner, Anna E. Kocsis, Krisztina Nagy, István Andó, András Dér

**Affiliations:** 1 Hungarian Research Network, Biological Research Centre, Institute of Biophysics, 6726 Szeged, Hungary; danielpetrovszki37@gmail.com (D.P.); kocsis.anna@brc.hu (A.E.K.); nagy.krisztina@brc.hu (K.N.); der.andras@brc.hu (A.D.); 2 Faculty of Science and Informatics, University of Szeged, 6720 Szeged, Hungary; zsomborkongorf@gmail.com (Z.F.); baumgartnermargareta@gmail.com (M.B.); biczok.brigi@gmail.com (B.B.); d.kira00@icloud.com (K.D.); kevind737@outlook.com (K.D.); berillkirner@gmail.com (B.B.K.); 3 Hungarian Research Network, Biological Research Centre, Institute of Genetics, 6726 Szeged, Hungary; ando.istvan@brc.hu

**Keywords:** label-free biosensor, biological cells, fiber optics, proof of concept, point of care, easy-to-use tool

## Abstract

Here, we report a rapid and accurate optical method for detecting cells from liquid samples in a label-free manner. The working principle of the method is based on the interference of parts of a conical laser beam, coming from a single-mode optical fiber directly, and reflected from a flat glass surface. The glass is functionalized by antibodies against the cells to be detected from the liquid sample. Cells bound to that surface modify the reflected beam, and hence, change the resulting interference pattern, too. By registering and interpreting the variation in the image, the presence of cells from the sample can be detected. As for a demonstration, cell suspensions from a U937 cell line were used in glass chambers functionalized by antibodies (TMG6-5 (mIgG1)) to which the cells specifically bind. The limit of detection (LOD) of the method was also estimated. This proof-of-concept setup offers a cost-effective and easy-to-use way of rapid and specific detection of any type of cells (including pathogens) from suspensions (e.g., body fluids). The possible portability of the device predicts its applicability as a rapid test in clinical diagnostics.

## 1. Introduction

Detection of cells from body fluids is traditionally accomplished in a microbiological laboratory, requiring incubators, skilled assistance, and sterile conditions for cell-culturing. Subsequently, cells are identified by some labeling techniques like ELISA [1]. Both steps are rather costly and time-consuming, while usually there is a significant time pressure for early diagnosis. On the contrary, label-free techniques, regardless of utilizing electrical or optical effects, generally do not need high-tech equipment, and normally can be applied on-site, albeit they are less sensitive [2,3,4,5,6,7,8,9].

The work presented here introduces a novel all-optical, label-free technique for recognizing and identifying cells from liquid samples. The method of detection is based on optical interference of parts of a laser beam. As a laser light mediator, a single-mode optical fiber was inserted into a fluidic channel of straight walls created from flat glass plates. Since the outcoupled beam is divergent, part of it is reflected from the flat glass substrate at the bottom of the channel, eventually meeting with the directly propagating, unreflected light, giving rise to interference, accordingly. Since any object close to the glass surface disturbs the beam reflected from it, a change in the interference can be observed upon adsorption of the analytes. e.g., cells attached to the reflecting bottom of the fluidic channel can bring forth a significant change in the interference pattern.

The specific identification of the cells is based on an antibody–antigen reaction. If, namely, the surface is functionalized by antibodies against a certain cell type, only that type of cell can be anchored to it [10,11]. To demonstrate the principle of detection and identification by our method, here we used the human U937 lymphocyte cell line and respective antibodies, while the results are discussed in terms of further potential general utilization [10].

On the whole, we established a simple and robust label-free method for the detection of cells from suspension. The operation principle of the method allows the rapid and cheap detection of biological objects, including pathogenic microorganisms from body fluids, while its simple and user-friendly implementation makes it a promising tool for point-of-care applications.

## 2. Materials and Methods

U937 cells from a human lymphatic model cell line were grown in a humidified, 37 °C incubator with 5% CO_2_, under normal cultivating conditions [12]. The cell counting of the stock suspension (3 × 10^6^ cells/mL) was performed via the traditional Bürker-chamber method, and its error was less than 5% [13]. Prior to starting the measurement procedure, a concentration series was prepared by successive dilutions of the stock suspension, with final concentrations of 10^6^, 3 × 10^5^, 10^5^, 3 × 10^4^, 10^4^, 3 × 10^3^, and 10^3^ cells/mL. As for the possible change in the cell concentrations during the measurement procedure, the total measuring time for the whole concentration series was about two hours. During the measurement, regarding the doubling time of these cells (48–72 h, under optimal conditions (e.g., constant, 37 °C temperature) [14], the increment—calculating with a pessimistic approach, assuming τ_double_ = 48 h doubling time and optimal culturing conditions—is less than 6%.

**Figure 1:**
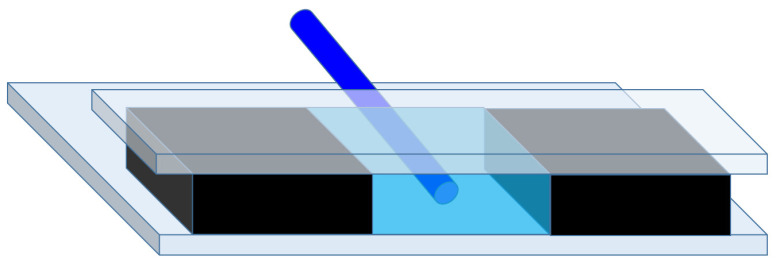
Schematic 3D figure of the device. The side blocks (black) and the glass plates on top and the bottom (gray) form the sample chamber. Thanks to the leveled end faces and edges of the blocks and glass plates the sample liquid (light blue) has a flat and vertical surface, which is at the same time the output optical window. The laser light enters the sample chamber from a single-mode optical fiber (dark blue). The figure is not to scale.

For the demonstration of the principles of the method, a fluidic chamber was built from microscope slides with two spacers in between. The schematic representation of the device is shown in Figure 1. The top slide is shorter, allowing an easy way to insert the optical fiber, and the inner surface is ground, in order to avoid too much light reflected back to the direct-light region. As spacers, slices of microscope slides that were a bit longer than the upper glass plate were used. Their thickness was around 1 mm, while the diameter of the stripped optical fiber was 125 µm. The top plate plays a dual role. It holds the liquid sample in the channel, and its edge maintains the liquid surface flat and perpendicular to the surface of the bottom plate by means of surface tension. Note that although both the back and the front ends of the channel are open, being the walls of the sample chamber hydrophilic, for such a height of the liquid layer (1 mm), the capillary forces keep the liquid in. This technique has already been utilized successfully for building a flat-ended optical waveguide out of a photopolymer liquid at the end of a single-mode optical fiber [15]. At the final assembly, a UV-curable optical adhesive (NOA81, ThorLabs, Newton, NJ, USA) and a transparent liquid were added between the parts, and the ending edges of the upper and lower glass plates were precisely aligned together. As for the final step, a light flash from a mercury arc lamp (of 100 W power for 5 s) was applied to cure the NOA81. The leveled ends of the glass plates—with the help of the surface tension—defined the end surface of the liquid with which the chamber is filled. The (stripped) optical fiber was pushed and fixed onto the bottom surface, ensuring its optical axis was parallel with it.

In order to bind the analyte particles (i.e., cells in this case) onto the surface of the sample chamber, it was functionalized with specific antibodies [11]. The assembled device was cleaned by ultrasonic treatment in Isopropanol (IPA), and then dried. The sample chamber was filled up with AnteoBindTM Biosensor (AnteoTech, Eight Mile Plains, Queensland, Australia) and incubated for 15 min at room temperature to facilitate antibody binding to the glass surface as a chelator. After that, the chamber was emptied, and flushed three times with Phosphate Buffer Saline (PBS). Subsequently, it was filled with the antibody (TMG6-5, mIgG1) solution and incubated for an hour at room temperature. Devices for control measurements were prepared in the same way, except for the antibody in the last step, which was IgG goat anti-mouse. The latter antibody covered the pretreated surface, but did not specifically bind the U937 cells.

A red laser light beam (RLT650-100MGS laser, Roithner LaserTechnic, Vienna, Austria, 658 nm, 100 mW output beam) was coupled into a single-mode optical fiber (ThorLabs Inc., Newton, NJ, USA, SM600, NA = 0.12). The chosen wavelength is one of the most common ones for cheap lasers, but the same principles used in this paper apply to other wavelengths in the visible, as well. Label-free biosensing is accomplished by the interference of two parts of a divergent laser light beam exiting a single-mode optical fiber. The lower part of this conical Gaussian measuring light beam hits the channel–substrate interface, and the vast majority of the intensity is reflected from it (for more details, see Supplementary Information, Figure S1, Equations (S1) and (S2)). The two coherent beams interfere, and the resulting interference pattern can be seen at the surface of a screen. When biological cells are attached to the surface, they modify the reflected light, which results in a variation in the interference pattern relative to the case without cells.

As the first step of the measurements, the measuring channel of the device, functionalized with antibodies specific to the human lymphatic cell line, was filled up with a liquid suspension of U937 cells, and the output interference pattern was recorded at regular intervals, to follow the process of sedimentation of the cells. After 15 min, the output pattern was not changing anymore, indicating that the sedimentation of the cells on the bottom surface was complete. The following 15 min were left for the formation of the antibody–cell binding. Then, the chamber was gently flushed with PBS three times, in order to remove the cells not anchored specifically to the antibodies. Eventually, the final interference pattern was recorded in PBS. For reference, the same procedure was repeated with a device functionalized with non-specific antibodies (IgG goat/anti-mouse). Special care had to be taken during the measurements, to avoid any air bubble(s) remaining in the sample chamber. On the one hand, these might prevent cell adhesion, while on the other, they distort the measuring laser beam, introducing artifacts in the interference pattern.

## 3. Results

### 3.1. Working Principle

**Figure 2:**
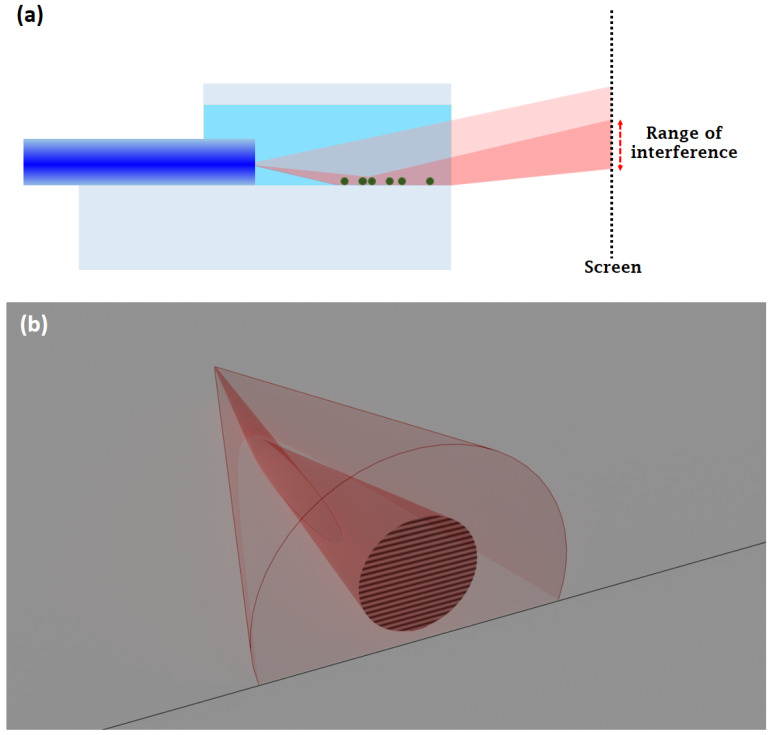
Schematic representation of the working principle: (**a**) Side view of the light path. The light red region shows the laser beam originated from the single-mode optical fiber, forming a coaxial light cone, and most of it (darker red) is reflected from the bottom surface of the sample-holding space (light blue), i.e., the interface functionalized by the analyte cells (green dots). In the overlapping area of the two parts, there occurs an interference that can be visualized/recorded by a screen (or a sensor of an imaging device). In order to make it easier to see the concept, the drawing in the figure is not to scale (actually the diameter of the optical fiber is 125 µm, while its distance from the end of the glass is about 4600 µm). (**b**) A 3D representation of the conical light beam. The color code is the same as used in (**a**). The lower part of the direct beam hits the bottom surface of the sample chamber in a parabolic region (shown in lighter red) and is reflected from it. Eventually, the direct and the reflected parts of the beam are stopped by the screen, where the interference is detected. The local change of reflectance in the elliptic region can be monitored as a variation in the interference stripes on the surface of the screen/detector. (The thin black line in the figure represents the edge of the substrate).

The light coming from the optical fiber is slightly divergent, having a cone shape, and Gaussian intensity distribution as a function of distance from the axis of the optical fiber (Figure 2). The numerical aperture (NA = 0.12) of the single-mode optical fiber determines the half-angle of that light cone. The exit end of the core of the optical fiber acts as a light source in this case, and it is centered at the half-diameter of the optical fiber (62.5 µm) from the surface of the glass plate. There is an area from where the incident light beam is reflected. Since the angle of the light cone is rather small (less than 5 degrees), obeying the optical laws, most of the light incident on the surface is reflected from it, and eventually meets the directly propagating light. Note that that some parts of the secondary reflected light from the lower surface of the glass substrate might also contribute to the interference to a lesser extent, but this does not represent a practical limitation of our method, since the evaluation procedure does not make use of the which-way information of the signal beam (for more details, see Supporting Information).

Due to geometrical reasons, the reflected light travels a longer way to a certain point on the screen than the directly propagating one, in the same medium. Since in this case, the light is a (coherent, monochromatic) laser beam, the differences in the optical pathlength result in an interference pattern (Figure 2). For this particular arrangement, it is a series of parallel bright stripes separated by dark ones (Figure 2b).

**Figure 3:**
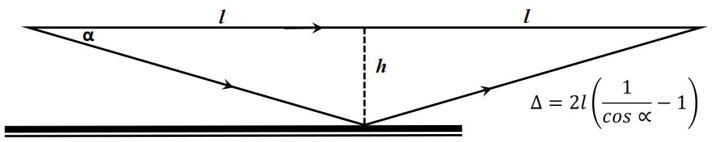
The optical path difference for a reflected and a direct ray.

According to the notations of Figure 3, Δ stands for the optical path difference, up to the distance 2*l*, between the reflected light leaving the optical fiber under angle *α*, and the one that comes directly in the axis. It can be calculated as:

(1) $$∆=2l\left(\frac{1}{cos\alpha }-1\right)$$

In the interference pattern, bright stripes occur when Δ is the multiple of λ = 673 nm, the wavelength of the light, and there are dark stripes in between, corresponding to a Δ of an odd multiple of the half-wavelength. If there is any disturbance in the reflected beam due to absorption or light scattering by particles on the surface, the interference lines become changed or distorted. In this way, any object of size comparable to or larger than the wavelength on the reflecting surface can be detected by observing the variation in the interference fringes. In our case, these objects are the cells attached to the functionalized surface of the glass plate.

The distance of the optical fiber should be adjusted carefully, so as to avoid its hitting the top surface of the sample chamber.

### 3.2. Evaluation Procedure

**Figure 4:**
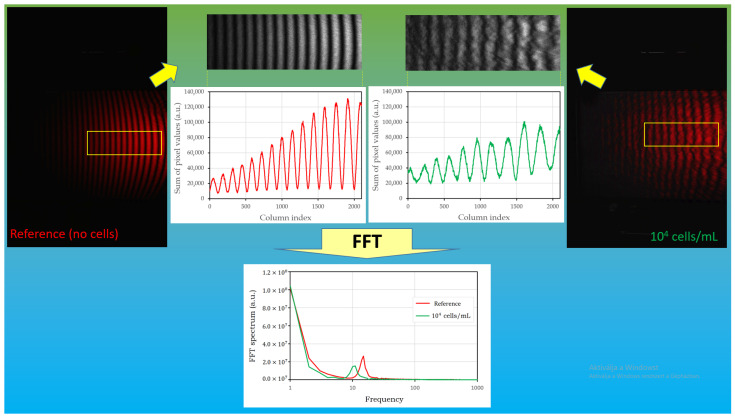
Schematic representation of the evaluation procedure. In the figure, “1E4” refers to 10^4^ cells/mL concentration used in the experiment, selected as an example to demonstrate the process (for details for all of the concentrations see Figure 5, Figure 6 and Figure S2 in the Supporting Information). Interference pattern of the reference (functionalized surface in pure PBS, without cells) labeled as “Reference” in the leftmost insert. Interference pattern recorded after the completed measuring cycle (after the cells bound to the surface and the chamber was flushed 3X with PBS) is in the rightmost insert. After a simple image processing procedure (see Figure S2, Supporting Information), represented by the middle panel, Fourier spectra were calculated for quantification of the effect.

At first, the reference experiment was carried out when the inner wall of the device was coated with non-specific antibodies. Having completed the measuring cycle (i.e., after final washing with pure PBS buffer), no cells were found to be attached to the surface of the channel, as checked by a microscope; however, the parallel stripes were recorded as a reference image when the channel was filled up with the buffer, shown in Figure 4. During the next experiments, another device functionalized by the specific antibodies was used with the same procedure. In this case, as a result of the adherence of cells to the lower surface of the fluidic channel, the interference pattern was found to be distorted (Figure 4). To quantify the difference, utilizing a MATLAB script (for details see Figure S2 and the script itself in the Supporting Information), we created a one-dimensional array by column-wise summarizing the values of the image pixels, yielding sinusoidal curves along the dimension perpendicular to the stripes as “reduced interferograms” (Figure 4, middle inserts). Subsequently, a Fourier transform was carried out, yielding the intensity distribution as a function of space frequency (Figure 4, bottom insert). The magnitude of the effects was then defined by the ratio of the amplitudes of the main peaks of the Fourier components determined from the interference fringes recorded at the end of the measuring cycle, and in the reference image. We found this method superior to other methods attempting to determine visibility.

### 3.3. Calibration of the Device

**Figure 5:**
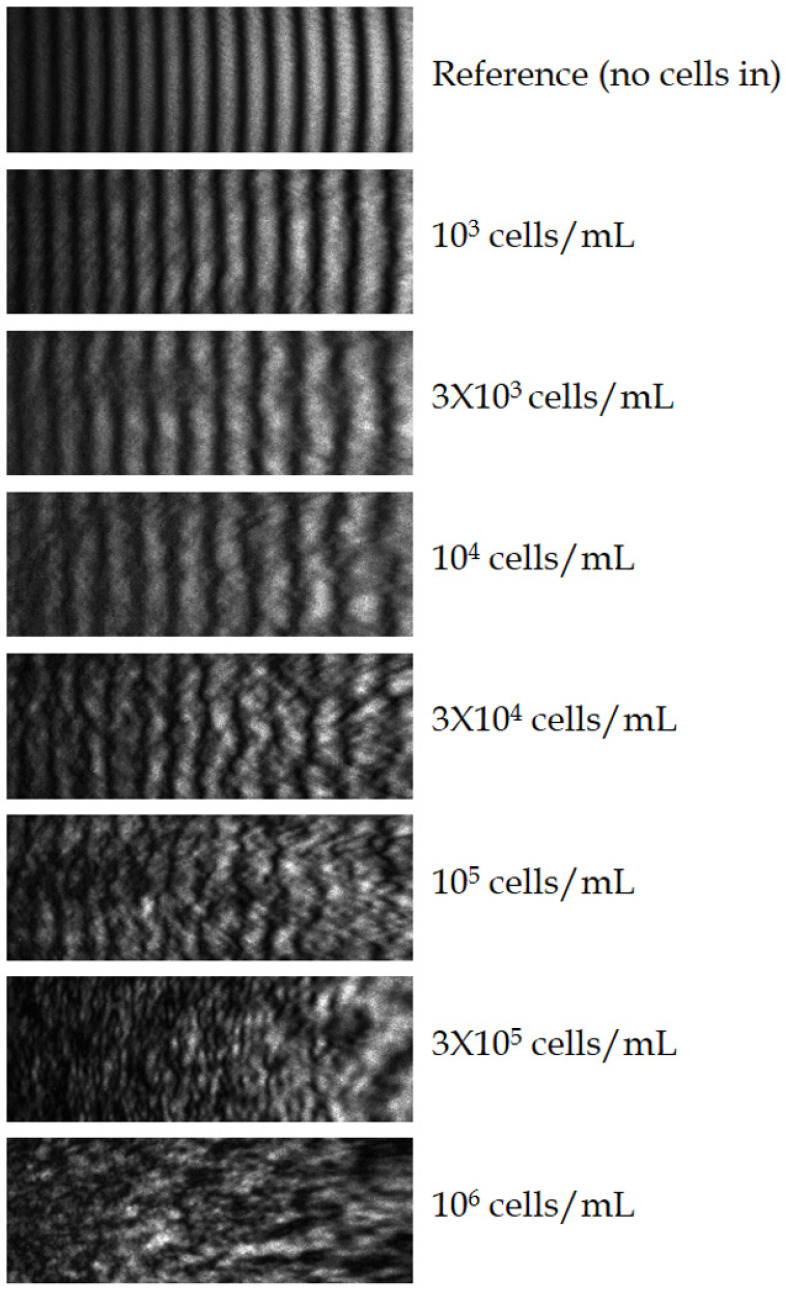
Grayscale images of the interference patterns recorded at various cell concentrations from 10^3^ to 10^6^ cells/mL, set in quasi-exponential-scale steps, and for the cell-free reference. (The resolution of the original images is 2100 × 750 pixels).

To determine the cell-concentration dependence of the method, a concentration series of cell suspension, from 10^3^ to 10^6^ cells/mL, was prepared, and the above procedure was carried out for each concentration (Figure 5).

Note that the frequency values assigned to the maxima of the main peaks might occasionally be shifted during the measuring cycle (see Figure S2, Supporting Information), by effects due to the possibly different meniscus curvatures at the beginning and the end of the measurement. However, such a virtual change in the magnification of the fringe pattern does not alter the intensity distribution among the stripes of the main component and the rest, so it does not influence the weights of the Fourier components, either.

**Figure 6:**
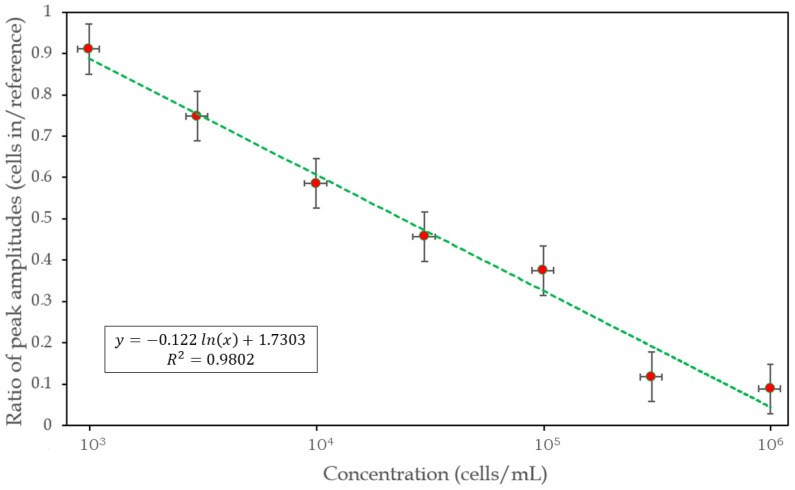
Cell-concentration dependence of the size of the effect, and a logarithmic fit to the 7 measured points (appearing as a straight line in the semi-logarithmic representation). The horizontal and vertical error bars represent a pessimistic error estimate based on the accuracy of the cell concentrations (for details, see Section 2), and 3 successive measurements per concentration, respectively.

The ratio of the main peak values of the sample and reference spectra, respectively (Table 1), depicted in a semi-logarithmic plot (Figure 6) shows a linear character in the examined concentration range, implying a logarithmic relationship between their signal size and cell concentration, apparently strictly following the Weber–Fechner law [16].

## 4. Discussion

The detected interference is determined by the reflectivity of the functionalized surface that depends on the number of cells attached to the bottom plate, irrespective of the sample volume they were sediment from. Hence, by increasing the sample volume, when possible, the LOD can be improved, practically at will. Since there is no need for a top wall for the measuring chamber, it can be extended upward to hold higher sample volumes. For example, the sample volume in our experiments was ca. 100 µL, but it can be easily expanded several-fold if there is enough sample available.

In such cases, however, where a lower sensitivity is sufficient (e.g., only the presence or absence of a microorganism is the question), conventionally available, horizontally aligned glass capillaries of a rectangular cross-section can also be used as measuring chambers (see also Supporting Information, Figure S3). In this case, reflections of the divergent beam take place from four adjacent walls, and a more complex, “chessboard-like” interference pattern is generated on the screen, in case of a clean, reference solution. However, having the cells (sediment) cover the bottom wall, the interference pattern is reduced to stripes (Figure S4). In this case, an evaluation software could apply either two 1D or one 2D Fourier transforms, to quantify the effect.

Since the device is a relatively small, handheld one, no special laboratory equipment and expertise are needed for performing a test [17,18]. Considering the easy transportability of the device, the method can be ideally suited for outdoor applications and point-of-care diagnostics [19,20].

## Figures and Tables

**Table 1:** The values plotted in Figure 6. The ratios are presented in four-digit precision showing the degradation and distortion of the interference pattern as the concentration of cells increases.

Concentration (Cells/mL)	Amplitude Ratio (Cells in/Reference)
10^3^	0.9105
3 × 10^3^	0.7479
10^4^	0.5857
3 × 10^4^	0.4570
10^5^	0.3749
3 × 10^6^	0.1172
10^6^	0.0888

## Data Availability

All related data are available from the corresponding author upon reasonable request.

## Supplementary Materials

The following supporting information can be downloaded at: https://www.mdpi.com/article/10.3390/bios14090421/s1. Figures S1 and S2: Details of the light path and the evaluation process; Figures S3 and S4: measurements with alternative cuvette geometries; Figure S5: Phase contrast image of the cells attached to the interface; Videos S1 and S2: Recordings of the changes of the interference patterns during sedimentation, at various cell concentrations.
