# Supplementary material for: Optical Interferometric Device for Rapid and Specific Detection of Biological Cells
Source: Biosensors (Basel). 2024 Aug 29;14(9):421. doi: 10.3390/bios14090421 (PMC11430435; doi:10.3390/bios14090421)
Supplement: Supplementary file 1 [file biosensors-14-00421-s001.zip › biosensors-3050400-supplementary.pdf]

Supporting Information

Fresnel's Equations

When a light wave arrives at a flat interface of two materials with different indexes of refraction, a part of it is reflected and the other part continues its way to the other side, but in a different direction (in other words, it is refracted):

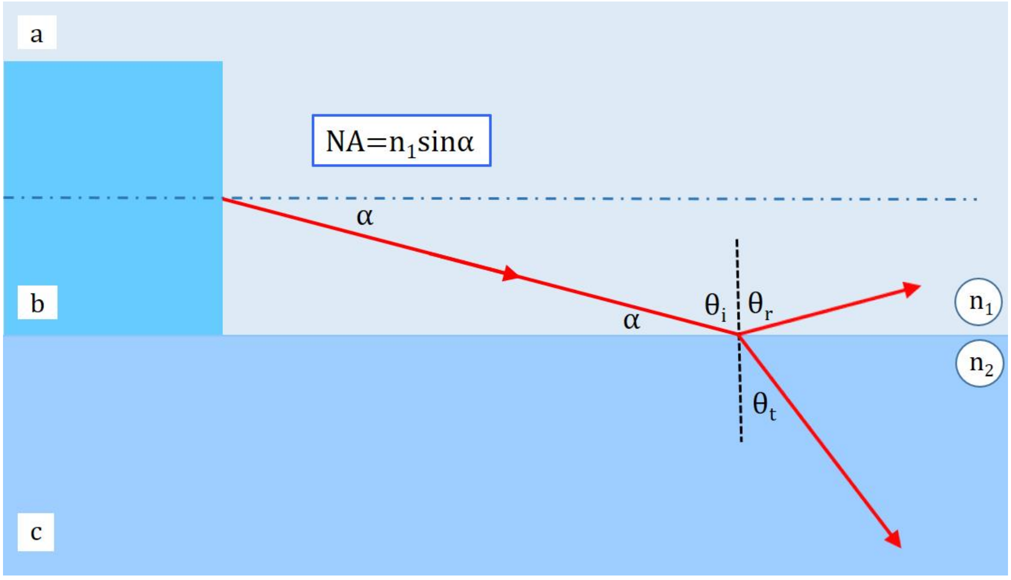

Figure S1. Ray representation of the light paths in the case when the light beam (red line) arrives from an optical fiber (b) into the material (a), and hits the surface of the other material (c) under the angle α, i.e., under the angle of incidence θ_i. The refracted part penetrates to the material (c) and, according to the corresponding refractive indices n_1 and n_2, under the transmitted angle θ_t.

The angle of incidence is the same as the angle of reflection ($\theta_i = \theta_r$) while the angle of refraction θ_t is governed by Snell's law.
The intensities (and amplitudes) of these two partial beams are described by Fresnel's equations. For both the *p* and *s* polarization (*p* stands for the ***E*** vector oscillating in the plane of incidence, while *s* for perpendicular to that).

$$r_s = \frac{n_1 \cos\theta_i - n_2 \cos\theta_t}{n_1 \cos\theta_i + n_2 \cos\theta_t},\ R_s = |r_s|^2 \text{ (S1)}$$

$$r_p = \frac{n_2 \cos\theta_i - n_1 \cos\theta_t}{n_2 \cos\theta_i + n_1 \cos\theta_t},\ R_p = |r_p|^2 \text{ (S2),}$$

where *r* refers to the amplitude and *R* to the intensity of the reflected light waves. Our laser source has a linearly polarized beam, and it was set to be s-polarized ("horizontal") where the reflection is the highest. Although the single-mode optical fiber we used was not a polarization-maintaining one, the beam coming out from it was mostly s-polarized.

Hence, the actual reflectivity was between the R_p and R_s (but closer to the latter).

In order to determine the pessimistic estimate of the reflectivity, substituting the refractive indices (n_1=1.333, n_2=1,51) and the highest angle corresponding to the nominal NA=0.12 of the optical fiber in water, considered at the beam waist (ca. 5°), the angle of incidence is 85°, from which we obtain R_s= 0.526, R_p=0.434. However, the laser beam from the optical fiber is a Gaussian beam, so most of the light energy propagates in the middle part, under even higher angles of incidence. For example, at θ_i=88°, we obtain R_s= 0.772, R_p=0.716, etc. So, it can be concluded that most of the intensity that was reflected from the first surface took part in forming the interference.

Summed vectors	FFT spectrum	Concentration	Summed vectors	FFT spectrum	Concentration
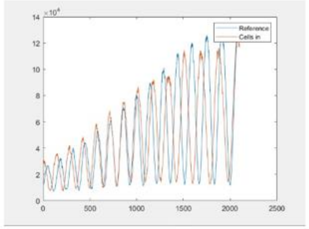	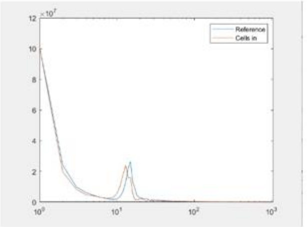	10^3 cells/mL	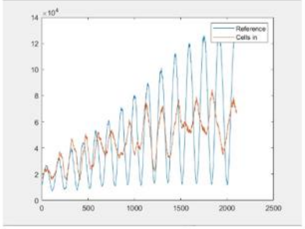	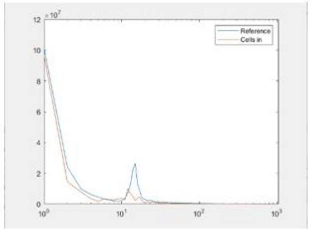	10^5 cells/mL
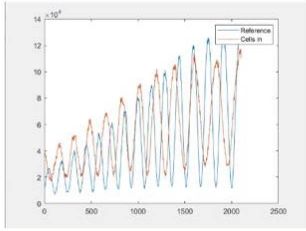	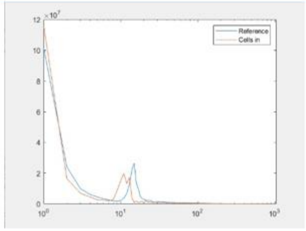	$3x10^3$ cells/mL	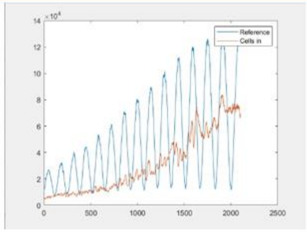	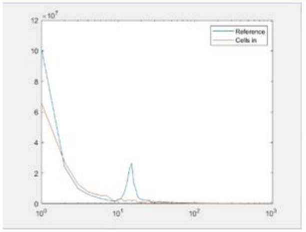	$3x10^5$ cells/mL
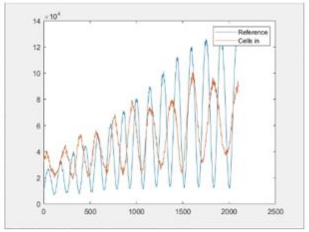	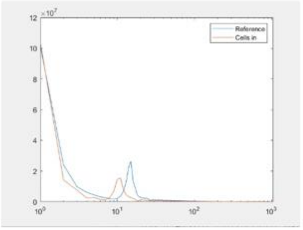	10^4 cells/mL	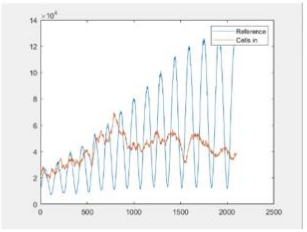	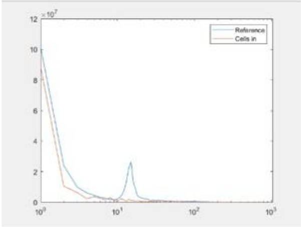	10^6cells/mL
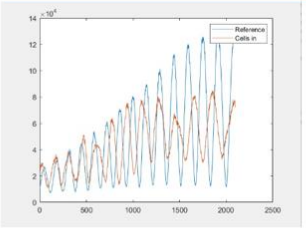	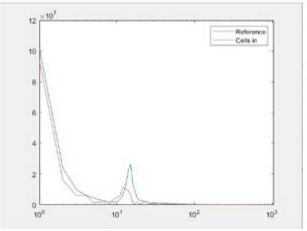	$3x10^4$ cells/mL			

Figure S2. On the chart, all the concentrations that were studied are presented. As has been described, color images were taken by a photo camera, and smaller parts were cropped for later analysis. At the end of the process for each concentration, a 2100-by-750 pixel size, 8-bit images were produced. A small MATLAB script was run and two graphs (and data series) were generated for each concentration. On the chart at the left, the vector is presented; it is an array of integers. Each number comes from adding up the pixel values of a column. This was performed for each column of the pixels of the input picture, and the result can be seen on the graph. The other graph next to that shows the amplitudes of the Fourier frequencies of that data array. Since there is a relatively large number of data points, a semi-logarithmic representation was chosen. In this way, all the data points (i.e., the whole spectrum) are presented, yet the important parts (peaks) at low frequencies can be well visualized. The default colors of MATLAB were used, the blue line represents the Fourier spectrum of the reference (with no cells) and the red one represents the sample with cells. Note, how the high amplitude waves in the left graphs (signs of clearly visible interference stripes in the image) disappear gradually as the cell concentration increases. This degradation is quantified by determining the ratio of the main peaks of the Fourier spectra.

The MATLAB script, used for the graphs is copied here as a text:

```
start=imread('ref_.JPG');
finish=imread('1e6_.JPG');
start_vector=sum(start(:,1:2100)); % reference vector
finish_vector=sum(finish(:,1:2100)); % 'cells in' vector
figure
plot([start_vector' finish_vector']) % graphs of the two vectors
legend('Reference','Cells in')
f_start=fft(start_vector); % FFT on control vector
f_finish=fft(finish_vector); % FFT on 'cells in' vector
len_start=length(start_vector); % length of the reference vector
len_finish=length(finish_vector); % length of the 'cells in' vector
ff_start=abs(f_start(1:len_start/2+1)); % Freq spectrum, reference vector
ff_finish=abs(f_finish(1:len_start/2+1)); % Freq spectrum, 'cells in' vector
max([ff_start(10:end)' ff_finish(10:end)']);
Peak_ref=max([ff_start(10:end)]) %MAx value over the 10 position in Reference
Peak_cells=max([ff_finish(10:end)])% Max value of sample over the 10 position
Peak_cells_in__per__Peak_ref=Peak_cells/Peak_ref % Ratio of peak amplitudes
figure % graph of the freq spectras (recalculated)
% The axis X is logaritmic
semilogx([abs(f_start(1:len_start/2+1)') abs(f_finish(1:len_finish/2+1)')])
legend('Reference','Cells in')
```

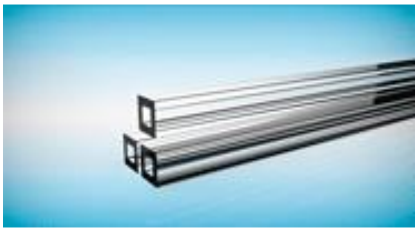

Figure S3. Rectangular capillaries [CM Scientific: VitroCom Glass Capillaries and Tubing, Square Capillaries].

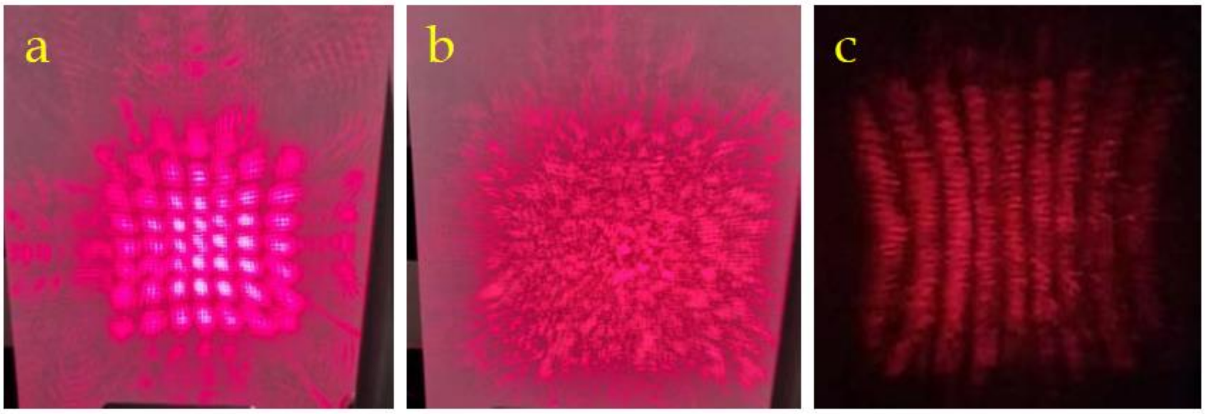

Figure S4. Interference fringes recorded in the case of rectangular channels, without cells (a), filled with cells during sedimentation (b), and when sedimented cells were attached to the bottom of the measuring cuvette (c). One can see that the horizontal part of the pattern has disappeared. Since the cells have covered (a part of) the bottom surface, and blocked the reflection from that only the interference fringes in the horizontal direction were affected (canceled), and this did not happen to the vertical walls and so did not influence the interference in the vertical direction.

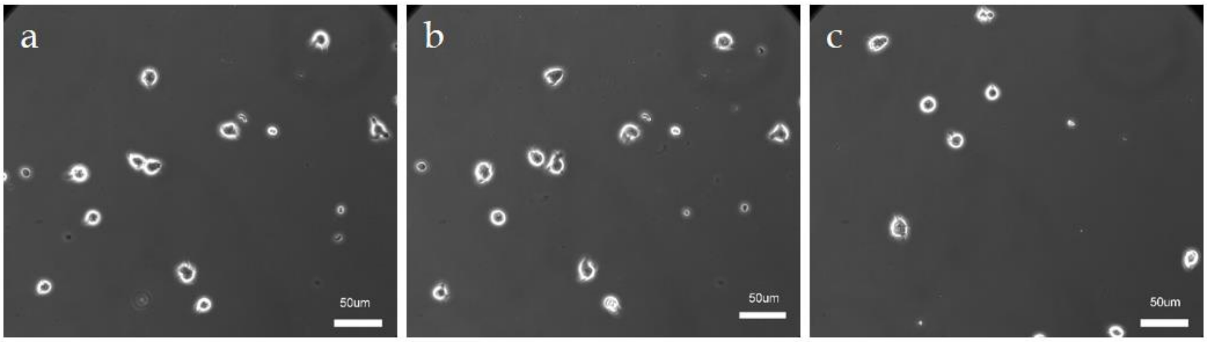

Figure S5. Phase contrast images (40X objective, Nikon Eclipse Ti) are about to study the cells attached on the surface. The S5/a shows the cells sedimented to the bottom surface, on the S5/b can be seen the cells attached after 30 minutes, and on the S5/c are the cells remained after washing.

Opportunity for increasing the Limit of Detection (LOD) of the method

As can be seen in the images of the interference fringes (lines), even a few cells make a detectable change. From a practical point of view, the device that we presented sort of 'counts' the cells present on the surface from where the light is reflected.
Let us suppose that there is a given output signal that belongs to 10 cells, and we have two different devices. Both of them have the same area of reflection but one of them has a five times higher sample volume (i.e., the sample chamber is five times higher).
According to the working principle, in this case, we will have the same output signal for the 10 cells, but, in the case of the device with a bigger volume, it belongs to a five times less concentration. This is true for all the concentrations including the one belonging to the LOD. In this example, the LOD of the bigger device is also less by the factor 5.
With proper modifications of the sample chamber, the volume, and hence the sensitivity, can be increased.

Videos:

V1. The recording of the interference patterns starts several seconds after the PBS in the sample chamber was replaced by the analyte (10^6 cells/mL). After some minutes, by the end of the video, the cells went down to the bottom, and the typical interference pattern (bent and dashed horizontal lines and a "grass-like" structure at the bottom) was about to be established.

V2. The interference pattern was recorded with a more dilute (10^4 cells/mL) analyte. The laser light is diffracted on the spherical individual cells, resulting in moving "bulls-eye" patterns that perturb the horizontal interference lines. Some of these moving circles seem to go to the top from the bottom: they are due to the light diffracted by the cells that are sinking in the direct beam that will be reflected from the bottom surface, and hence we see the mirrored image at the output. Here, it can be seen that only a few cells were present in the sample chamber, and their dynamic effect on the interference pattern was still noticeable.
